# Predictors of Increased Risk of Hepatocellular Carcinoma in Patients with Type 2 Diabetes

**DOI:** 10.1371/journal.pone.0158066

**Published:** 2016-06-30

**Authors:** Won Keun Si, Jung Wha Chung, Junhyeon Cho, Joo Yeong Baeg, Eun Sun Jang, Hyuk Yoon, Jaihwan Kim, Cheol Min Shin, Young Soo Park, Jin-Hyeok Hwang, Sook-Hyang Jeong, Nayoung Kim, Dong Ho Lee, Soo Lim, Jin-Wook Kim

**Affiliations:** 1 Department of Medicine, Seoul National University Bundang Hospital, Seongnam, Republic of Korea; 2 Department of Internal Medicine, Seoul National University College of Medicine, Seoul, Republic of Korea; The University of Tokyo, JAPAN

## Abstract

Epidemiological data indicate that type 2 diabetes is associated with increased risk of hepatocellular carcinoma (HCC). However, risk stratification for HCC has not been fully elucidated in diabetic population. The aim of this study was to identify potential predictors of HCC in diabetic patients without chronic viral hepatitis. A cohort of 3,544 diabetic patients without chronic viral hepatitis or alcoholic cirrhosis was established and subjects were randomly allocated into a derivation and a validation set. A scoring system was developed by using potential predictors of increased risk of HCC from the Cox proportional hazards model. The performance of the scoring system was tested for validation by using receiver operating characteristics analysis. During median follow-up of 55 months, 36 cases of HCC developed (190 per 100,000 person-years). The 5- and 10-year cumulative incidences of HCC were 1.0%, and 2.2%, respectively. Multivariate Cox regression analysis showed that age > 65 years, low triglyceride levels and high gamma-glutamyl transferase levels were independently associated with an increased risk of HCC. DM-HCC risk score, a weighted sum of scores from these 3 parameters, predicted 10-year development of HCC with area under the receiver operating characteristics curve of 0.86, and discriminated different risk categories for HCC in the derivation and validation cohort. In conclusion, old age, low triglyceride level and high gamma-glutamyl transferase level may help to identify individuals at high risk of developing HCC in diabetic patients without chronic viral hepatitis or alcoholic cirrhosis.

## Introduction

Hepatocellular carcinoma (HCC) is the sixth most common cancer worldwide, with more than 700,000 new cases annually [1]. The mortality of HCC is high, ranking third as a cause of cancer-related death [1]. To ensure early diagnosis and thus reduce HCC-related mortality, surveillance program has been advocated in populations at an increased risk of HCC [2, 3]. Liver cirrhosis and chronic viral hepatitis are well-known risk factors for HCC [2, 4], and surveillance for HCC has been recommended in these conditions [2, 5]. In addition, various other chronic liver diseases such as autoimmune, metabolic, and alcoholic liver disease also confer an increased risk of HCC [2, 3, 5, 6].

Substantial epidemiological data indicate that diabetes mellitus increases the risk of HCC. Several case-control [7–10] and cohort studies [11–16] have reported a positive association between diabetes and HCC, and recent meta-analyses have also shown that diabetes is independently associated with HCC [17–20]. However, the relative risk of HCC associated with diabetes is low (1.20–2.31) [12, 13, 15, 19, 21] compared with those of chronic viral hepatitis or alcoholic cirrhosis [22]. Considering the high prevalence of diabetes in general population [23], it would not be cost-effective to screen the entire diabetic population for HCC. Therefore, it would be clinically relevant to identify potential candidates for HCC surveillance in diabetes by stratifying the risk of HCC. However, studies investigating the predictors of enhanced HCC risk in diabetic patients are limited. Thus, the aim of this study was to identify potential predictors of HCC in patients with diabetes who do not have viral hepatitis.

## Materials and Methods

### Study population

This single-center, retrospective cohort study recruited patients aged ≥ 18 years with Type 2 diabetes who had visited Seoul National University Bundang Hospital (SNUBH), a tertiary referral center located at Seongnam, Republic of Korea, from May 2003 to April 2014 and been followed up for > 12 months. All consecutive patients were identified in the electronic clinical data warehouse (CDW) of SNUBH [24], and clinical and laboratory data were retrieved from the electronic medical record system (BESTCare) [24, 25]. Diagnosis of fatty liver was made by ultrasonography.The exclusion criteria were: (1) positivity for the hepatitis B surface antigen, (2) positivity for antibody to the hepatitis C virus, (3) alcoholic cirrhosis, (4) autoimmune liver diseases (autoimmune hepatitis, primary biliary cirrhosis) and (5) HCC either before or within 12 months of enrollment. This study was approved by the internal review board and ethics committee of SNUBH (B-1508/310-111). Informed consents were waivered due to observational nature of study. Patient records / information was anonymized and de-identified prior to analysis.

### Definitions

Type 2 diabetes was defined based on the definition of American Diabetes Association [26]. Liver cirrhosis was diagnosed using a combination of clinical, endoscopic, radiological, and histological assessment. Alcoholic cirrhosis was diagnosed when the liver cirrhosis was combined with a documented history of alcohol abuse or dependence, or with an alcohol consumption > 24 g/day in men or > 12 g/day in women, respectively, and without any other specific causes of cirrhosis [27]. Diagnosis of HCC was based on the guidelines published by the American Association for the Study of Liver Diseases [2]. The HCC was staged using the TNM staging system of the American Joint Committee on Cancer. FIB-4 was calculated as reported previously in order to estimate the stage of liver disease, using the following formula: age (years) × AST [IU/L]/(platelets [10^9^/L] × (ALT [IU/L])^1/2^ [28–30].

### Statistical analysis

The study subjects were randomly allocated either into derivation cohort or validation cohort by using R package ‘randomizr’ (https://cran.r-project.org/web/packages/randomizr/index.html). The Kaplan–Meier method was used to estimate the incidence of HCC during follow-up. The derivation cohort was used to build a Cox proportional hazards model predicting development of HCC. Independent predictors of HCC were identified from this model, and a scoring system was developed by incorporating relative risks identified from logistic regression analysis. The performance of this scoring model for predicting HCC development was tested by receiver operating characteristics (ROC) analysis.

Variables are reported as mean ± standard deviation, median with range, or number (%), as appropriate. Differences among continuous and categorical variables were analyzed using the Student’s *t*-test and the chi-squared test, respectively. Analyses were performed using IBM SPSS Statistics for Windows (version 22.0; IBM Corp., Armonk, NY, USA). A *p*-value of < 0.05 was considered statistically significant.

## Results

### Patient characteristics and HCC development

**Fig 1:**
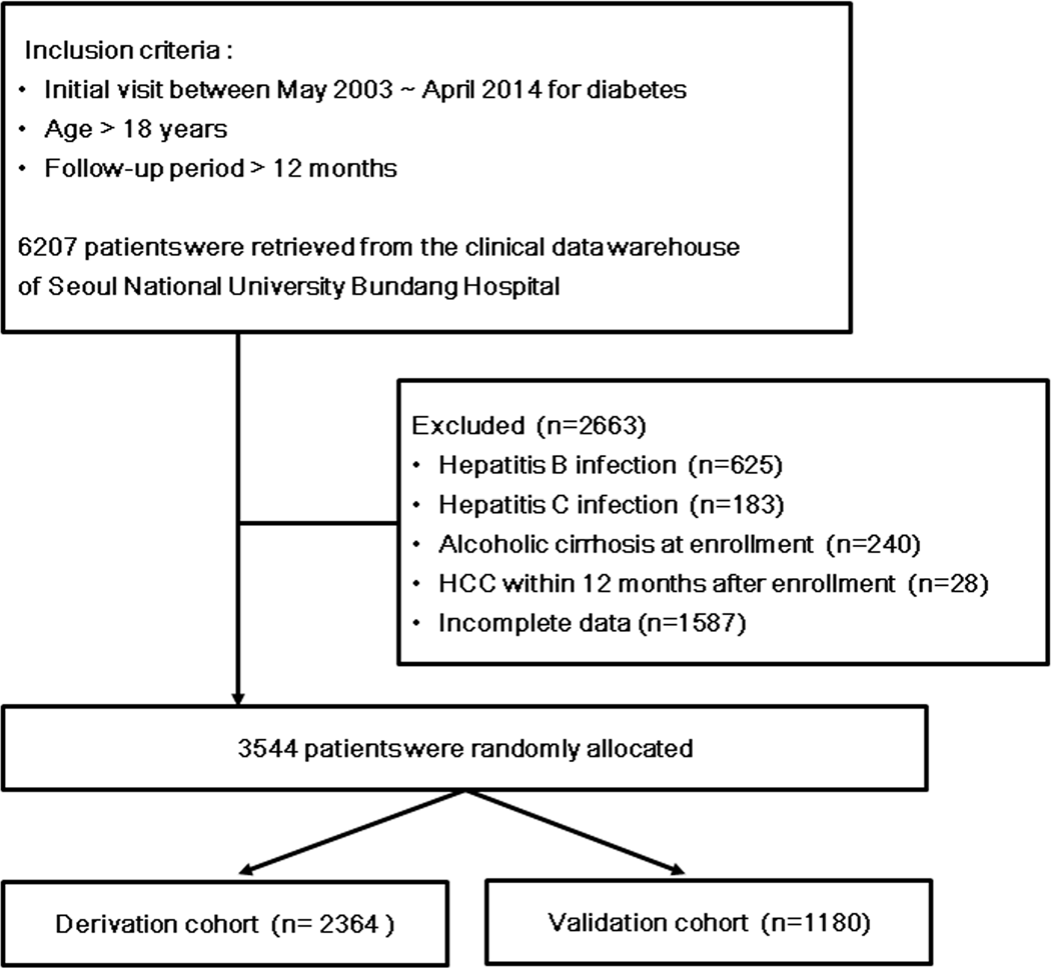
Study flow diagram. Diabetic patients without chronic viral hepatitis or alcoholic cirrhosis were identified and randomly allocated to derivation or validation cohort.

A total of 6,207 patients who met the inclusion criteria were identified. After excluding patients with chronic viral hepatitis, alcoholic cirrhosis, HCC before 12 months of follow-up and incomplete laboratory date, 3,544 patients were included in the final analysis (Fig 1). The study subjects were randomly assigned to derivation cohort (n = 2,364) and validation cohort (n = 1,180) for further analysis. There were no significant differences in the baseline characteristics between the two cohorts except for history of heavy drinking (7.7 vs. 5.8%, *P* = 0.038) (Table 1). During the study period of 12–146 months (median 55 months), metformin, sulfonylurea, DPP-4 inhibitor, alpha-glucosidase inhibitor, thiazolidinedione and insulin were prescribed in 73%, 50%, 37%, 17%, 15% and 54% of patients, respectively.

**Table 1:** Baseline and follow-up characteristics of derivation and validation cohorts.

Baseline characteristic	Derivation cohort (N = 2364)	Validation cohort (N = 1180)	*P*-value
Age, years	67 (24–101)	67 (21–94)	0.368
Male sex	1539 (65.1)	750 (63.6)	0.366
Duration of diabetes, years	6.6 (1–39)	6.6 (1–46)	0.973
Follow-up, months	57 (12–146)	58 (12–145)	0.128
Heavy drinking^†^	181 (7.7)	68 (5.8)	0.038
Fatty liver	1267 (53.6)	606 (51.4)	0.212
DM medication*			
Duration, months	38 (0–132)	39 (0–131)	0.512
Metformin	1748 (73.9)	876 (74.2)	0.850
Insulin therapy	1233 (52.2)	617 (52.3)	0.941
HbA1C (%)	7.1 (4.6–16.7)	7.2 (4.5–16.4)	0.699
Albumin (g/dL)	4.3 (1.8–5.3)	4.3 (2.3–5.5)	0.660
Bilirubin (mg/dL)	0.8 (0.2–5.2)	0.8 (0.2–13.3)	0.118
Cholesterol (mg/dL)	184 (64–555)	187 (63–474)	0.254
TG (mg/dL)	134 (26–1988)	133 (23–1672)	0.398
ALP (IU/L)	72 (24–1056)	72 (7–896)	0.165
AST (IU/L)	23 (5–874)	23 (3–658)	0.751
ALT (IU/L)	25 (3–1420)	25 (2–377)	0.781
GGT (IU/L)	33 (6–2060)	32 (5–2236)	0.377
Platelet (x10^9^/L)	231 (21–1198)	229 (6–792)	0.703
FIB-4	1.33 (0.20–74.55)	1.35 (0.20–58.38)	0.664
APRI	0.36 (0.03–15.8)	0.38 (0.03–20.0)	0.640

Variables are expressed as median (range) or n (%).

^†^ Alcohol consumption > 24 g/day in men or > 12 g/day in women

* Note that percentages are inclusive and add up to greater than 100%.

Abbreviation: TG, triglyceride; ALP, alkaline phosphatase; AST, aspartate transferase; ALT, alanine transferase; GGT, Gamma-glutamyl transferase; APRI, AST-to-platelet ratio index.

**Fig 2:**
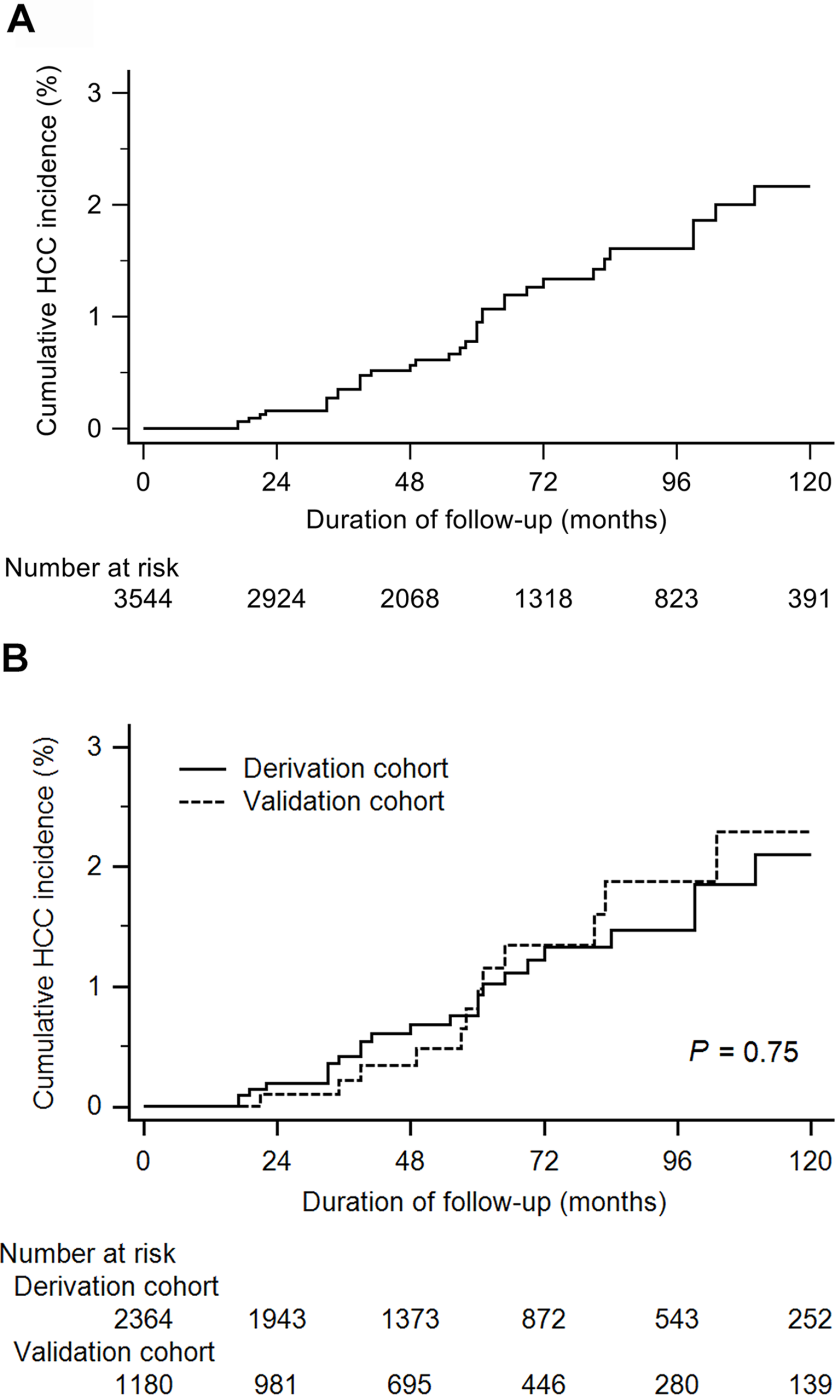
Cumulative incidence of hepatocellular carcinoma in overall diabetic patients without chronic viral hepatitis or alcoholic cirrhosis. (A) The 5- and 10-year cumulative incidences of HCC were 1.0% and 2.2%, respectively. (B) The incidences of HCC were similar between the derivation and validation cohort.

During the follow-up period, 36 patients developed HCC: 23 in the training cohort and 13 in the validation cohort. The overall incidence of HCC was 190 per 100,000 person-years. The 5-, and 10-year cumulative incidences of HCC were 1.0% and 2.2%, respectively (Fig 2A). The incidences of HCC were similar between the training and validation cohorts (*P* = 0.75; Fig 2B). The characteristics of these HCC patients are described in S1 Table. Age > 65 years, heavy drinking, presence of fatty liver, insulin therapy, low triglyceride (TG) level, high alkaline phosphatase, high aspartate aminotransferase, high gamma-glutamyl transferase (GGT), low platelet counts, high FIB-4 scores and high APRI values were associated with HCC development in derivation cohort. In validation cohort, age, insulin therapy, low albumin, high GGT, low platelet counts and high FIB-4 scores were associated with HCC development (Table 2).

**Table 2:** Comparisons of baseline characteristics in patients with and without subsequent development of hepatocellular carcinoma.

Variables	Derivation cohort (N = 2364)			Validation cohort (N = 1180)		
	HCC(n = 23)	No HCC(n = 2341)	*P* value*	HCC(n = 13)	No HCC(n = 1167)	*P* value*
Age > 65 years	21 (91.3)	1265 (54.0)	0.001	13 (100)	650 (55.7)	0.001
Male sex	19 (82.6)	1520 (64.9)	0.077	11 (84.6)	739 (63.3)	0.113
Heavy drinking^†^	5 (21.7)	176 (7.5)	0.011	2 (15.4)	66 (5.7)	0.134
Fatty liver	5 (21.7)	1262 (53.9)	0.002	6 (46.2)	600 (51.4)	0.785
Anti-HBs positivity	19 (82.6)	1599 (70.7)	0.255	6 (46.2)	806 (71.6)	0.061
Duration of diabetes, years	7.8	6.6	0.184	9.6	6.5	0.133
DM medication**						
Metformin	18 (78.2)	1730 (73.9)	0.812	11 (84.6)	865 (74.1)	0.390
Insulin therapy	18 (78.2)	1215 (51.9)	0.012	12 (92.3)	605 (51.8)	0.004
HbA1C > 7.1%	9 (52.9)	1066 (47.0)	0.624	4 (30.8)	555 (49.1)	0.189
Albumin < 4.0 g/dL	6 (31.6)	425 (18.6)	0.148	7 (63.6)	204 (18.0)	0.001
Bilirubin > 0.8 mg/dL	10 (52.6)	952 (41.6)	0.330	4 (33.3)	476 (41.9)	0.551
Cholesterol < 200 mg/dL	16 (84.2)	1450 (62.9)	0.055	9 (75.0)	711 (62.1)	0.359
TG < 150 mg/dL	20 (87.0)	1389 (59.3)	0.007	10 (76.9)	684 (58.6)	0.182
ALP > 80 IU/L	12 (63.2)	809 (35.3)	0.012	6 (54.5)	410 (36.1)	0.204
AST > 40 IU/L	7 (36.8)	299 (13.0)	0.002	2 (16.7)	158 (13.8)	0.779
ALT > 40 IU/L	6 (31.6)	528 (22.1)	0.373	3 (25.0)	265 (23.2)	0.882
GGT > 40 IU/L	18 (78.3)	902 (38.5)	0.001	10 (76.9)	449 (38.5)	0.005
Platelet < 120 x10^9^/L	2(11.8)	58 (2.7)	0.022	2 (18.2)	29 (2.7)	0.002
FIB-4 > 2.67	7 (41.2)	204 (9.4)	0.001	5 (45.5)	114 (10.6)	0.004
APRI > 0.7	9 (39.1)	176 (7.5)	0.001	3 (23.1)	98 (8.4)	0.09

Variables are expressed as n (%).

* Comparison between HCC and No HCC groups.

** Note that percentages are inclusive and add up to greater than 100%.

^†^ Alcohol consumption > 24 g/day in men or > 12 g/day in women

Duration of diabetes was not significantly different between HCC and no HCC groups. Anti-HBs positivity was similar between HCC and no HCC groups (Table 2). Anti-HBc was checked in a limited numbers of patients (n = 38), and the positive rates were similar between HCC and no HCC groups (*p* = 1.00).

### Predictors of increased HCC risk

A univariate analysis showed that development of HCC was significantly associated with old age, heavy drinking, low serum TG levels, high alkaline phosphatase levels, high AST levels, high GGT levels, low platelet counts, high FIB-4 score and high APRI score. Multivariate Cox regression analysis showed that age > 65 years (hazard ratio = 6.8; 95% CI, 1.5–30.1), low TG levels < 150 mg/dL (hazard ratio = 6.7; 95% CI, 1.5–30.1) and high GGT levels > 40 IU/L (hazard ratio = 10.1; 95% CI, 2.7–37.6) were independent risk factors for HCC (Table 3).

**Table 3:** Predictors of HCC by Cox proportional hazard model in derivation cohort.

Variables	Univariate		Multivariate	
	HR (95% CI)	*P* value	HR (95% CI)	*P* value
Age > 65 (years)	7.17 (1.68–30.61)	0.008	6.79 (1.53–30.07)	0.012
Male	2.74 (0.93–8.07)	0.067		
Heavy drinking^†^	3.28 (1.22–8.83)	0.019		
Fatty liver	0.47 (0.12–1.82)	0.548		
Metformin	0.86 (0.32–2.31)	0.758		
Insulin	2.64 (0.98–7.14)	0.055		
HbA1C > 7.1%	1.17 (0.45–3.04)	0.744		
Albumin < 4.0 g/dL	2.08 (0.79–5.48)	0.137		
Bilirubin > 0.8 mg/dL	1.58 (0.64–3.89)	0.318		
TG < 150 mg/dL	4.61 (1.37–15.51)	0.014	6.72 (1.50–30.06)	0.013
ALP > 80 IU/L	3.23 (1.27–8.20)	0.014		
AST > 40 IU/L	4.13 (1.62–10.50)	0.003		
ALT > 40 IU/L	1.54 (0.59–4.05)	0.382		
GGT > 40 IU/L	6.36 (2.36–17.15)	0.001	10.08 (2.71–37.55)	0.001
Platelet < 120 x10^9^/L	5.85 (1.34–25.62)	0.019		
FIB-4 > 2.67	7.20 (2.74–18.92)	0.001		
APRI > 0.7	8.88 (3.83–20.56)	0.001		

Abbreviation: CI; confidence interval.

^†^ Alcohol consumption > 24 g/day in men or > 12 g/day in women.

### Correlation between serum triglyceride levels and serum hepatic fibrosis marker

Since low serum TG level was a significant predictor of HCC development in our cohorts, we sought to know whether low TG levels were associated with hepatic fibrosis in diabetes. Pearson's correlation analysis showed that serum TG levels negatively correlated with FIB-4 scores in both cohorts (S1 Fig), suggesting that serum TG levels may be a surrogate marker of hepatic fibrosis in diabetes.

### Performance of HCC risk score for the prediction of HCC development in diabetes

**Fig 3:**
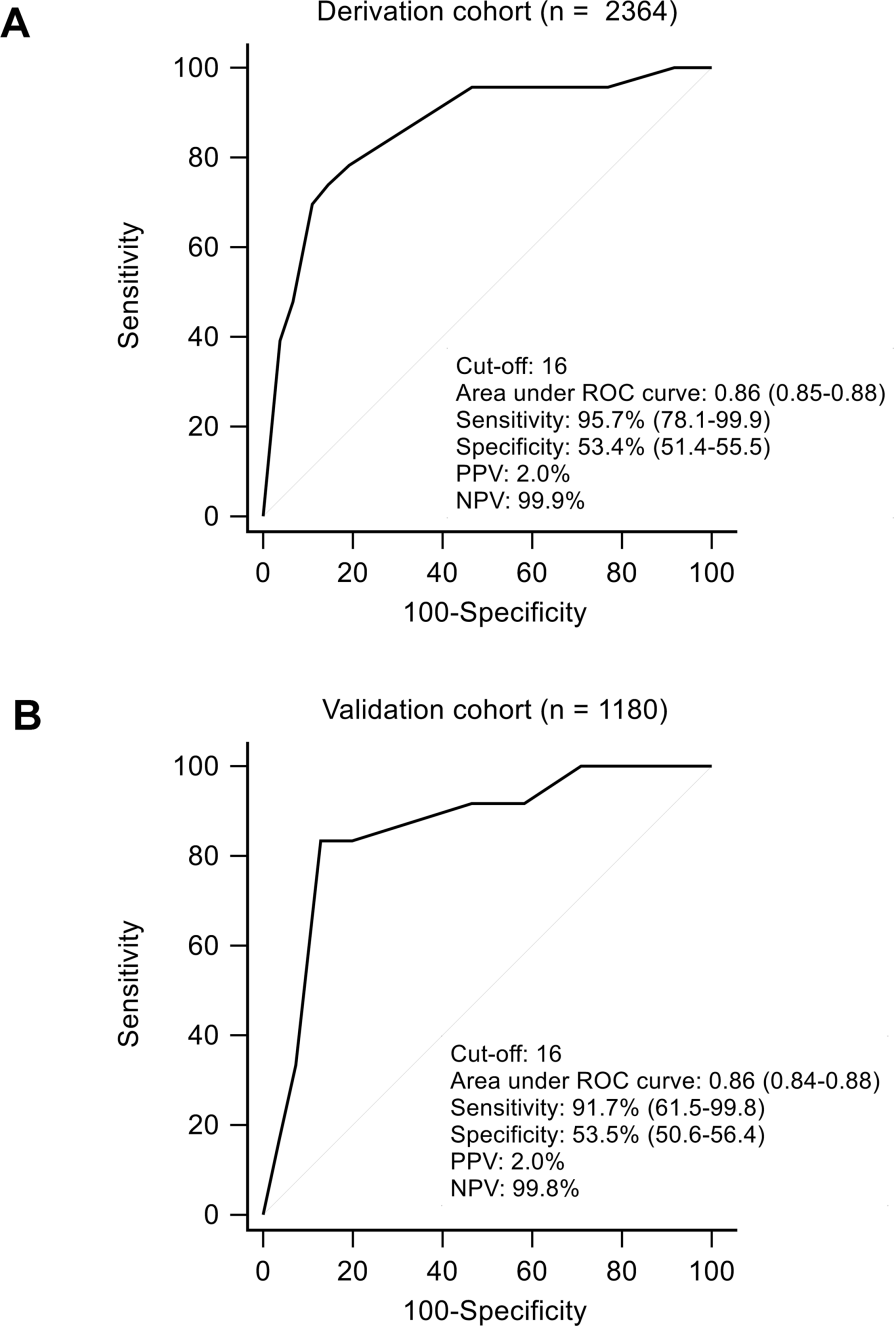
ROC analysis of the DM-HCC risk score in predicting development HCC in 10 years. At the cutoff value of 16, the area under ROC curve was 0.86 (95% CI, 0.85–0.88) in derivation cohort (A) and 0.86 (95% CI, 0.84–0.88) in validation cohort (B). The sensitivity, specificity positive predictive value and negative predictive value were shown with 95% confidence intervals in parentheses.

**Fig 4:**
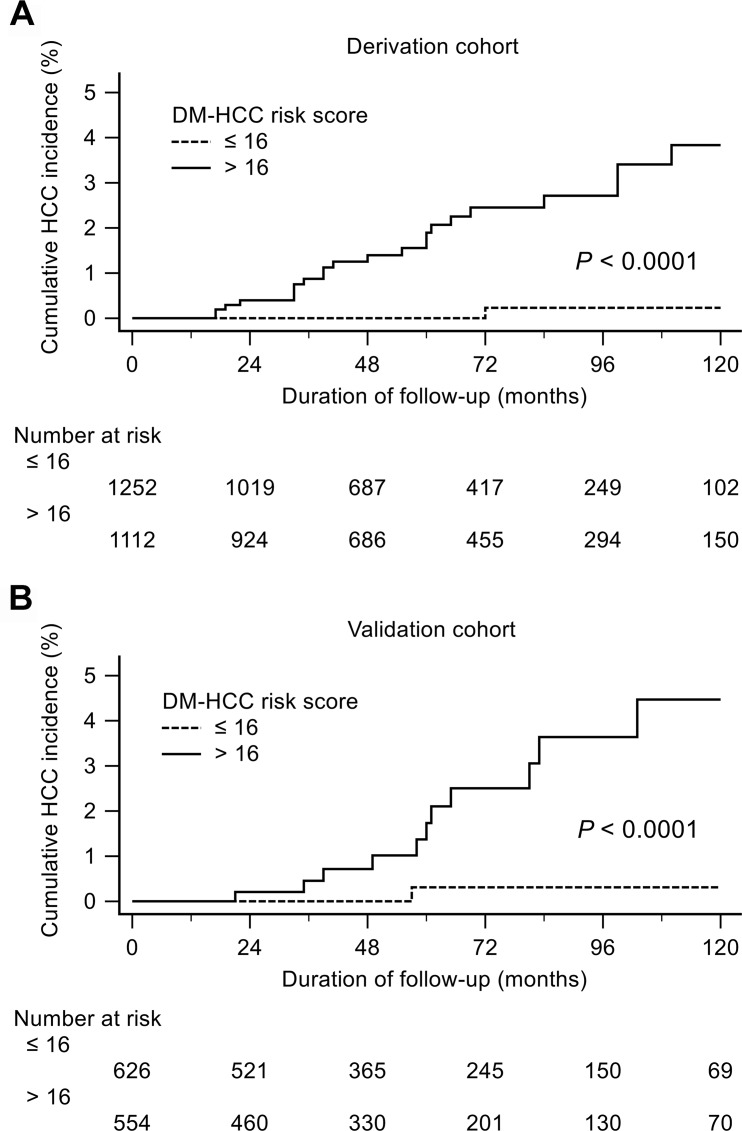
Cumulative incidence of hepatocellular carcinoma in diabetic patients with DM-HCC risk scores. Cumulative incidence of hepatocellular carcinoma in diabetic patients with low (≤16) and high (>16) DM-HCC risk scores in the derivation cohort (A) and the validation cohort (B).

A risk scoring system was developed from the results of multivariate analyses. The scores for each parameter (age, TG and GGT) were the rounded quotient of corresponding estimated coefficient from logistic regression analysis model, and the weighted sum of this DM-HCC risk score ranged from 0 to 33 (S2 Table). At the cutoff value of 16, the sensitivity, specificity and area under the ROC curve of DM-HCC risk score in the prediction of 10-yr HCC risk was 95.7%, 53.4% and 0.86, respectively, in derivation cohort, and 91.7%, 53.5% and 0.86, respectively, in validation cohort (Fig 3). The 10-year cumulative risk of HCC were 0.2% vs. 3.8% in patients with baseline DM-HCC risk scores ≤ 16 vs. > 16, respectively (*p* < 0.001) in the derivation cohort, and 0.3% vs. 4.5%, respectively in the validation cohort (*p* < 0.001) (Fig 4)

## Discussion

Accumulating evidence indicates that diabetic patients are at higher risk of HCC. However, the relative risk is modest, being around a two-fold increase in the cumulative probability of HCC [17–20]. Considering the high prevalence of diabetes in the general population, it is essential to identify predictors of HCC among patients with diabetes in order to define high-risk subgroups, as well as to develop an effective surveillance program in the diabetic population. To the best of our knowledge, little is known about the stratification of HCC risk in diabetic population. In this study, we found out that old age, low serum TG levels and high serum GGT levels were independent risk factors for HCC in a large retrospective cohort of diabetic patients without chronic viral hepatitis.

The incidence of HCC in our cohort was 190 per 100,000 person-years, which was higher than in a recent American cohort (24 per 100,000 person-years) [12], and slightly higher than in a Taiwanese cohort (118 per 100,000 person-years) [13]. This high incidence of HCC may be ascribed to the high median age of our cohort, considering the fact that age was a significant risk factor for HCC in our patients as well as in other cohort studies [12, 13]. Indeed, the HCC incidence of old-age population in the Taiwanese cohort was very similar to our result (142–274 per 100,000 person-years) [13].

The underlying mechanism of the association between low TG levels and increased risk of HCC in our study are not clear. Several studies have reported that TG levels decrease in advanced liver cirrhosis [31, 32], and TG levels may be negatively correlated with hepatic fibrosis in non-alcoholic steatohepatitis [33]. Our data also showed significant correlation between TG levels and serum fibrosis marker levels. Low TG levels, along with low platelet counts and high serum fibrosis marker levels, may indicate significant baseline liver injury in our cohort and TG may have remained significant in multivariate modelling as a more robust predictor of HCC compared to other fibrosis predictors. However, this hypothesis needs to be elucidated in future prospective studies.

It is intriguing that high serum GGT levels were associated with increased HCC risk. GGT levels frequently rise after prolonged alcohol consumption [34], may reflect advanced disease stages in fatty liver disease [35, 36], and also serve as a marker of HCC [37, 38]. Therefore, GGT may indicate HCC-predisposing condition such as prolonged alcohol consumption, advanced fibrosis or cirrhosis in diabetes. Since fatty liver disease is commonly associated with diabetes, it might be postulated that elevated GGT reflects fatty liver disease-associated cirrhosis in our study. The low baseline platelet counts and high baseline FIB-4 scores in our HCC patients (median = 3.3; reported cutoff for cirrhosis = 2.9–3.6) also suggest presence of advanced fibrosis in this group [28–30, 39]. Again, this explanation needs validation by further studies.

Alcoholic cirrhosis has high probability of HCC development [4, 40], and several studies have reported synergic effect of heavy alcohol consumption and diabetes in HCC development [41, 42]. Heavy drinking was more frequent in the HCC group in our study, but was not an independent risk factor for HCC development by multivariate analysis. One explanation is that our study may have underestimated the contribution of alcohol to actual HCC incidence in diabetes, because alcoholic cirrhosis was systematically excluded in our cohort. Alternatively, heavy drinking may have multicollinearity with elevated GGT levels and thus excluded in the multivariate analysis.

The unique feature of our study is that predictors of HCC development were derived from a large cohort of diabetic patients observed for a prolonged period of time. Although retrospective in nature, our cohort utilized established electronic data warehouse system [24, 43] to minimize selection bias, an approach commonly employed in both population- and hospital-based cohort studies [44–46]. In addition, the study population was randomized into derivation and validation cohorts, and the results from multivariate regression analysis were reproduced in the validation cohort.

This study had limitations. Firstly, it was a study from a single referral center, with relatively old age of study population. We enrolled more than 3,000 consecutive diabetic patients in order to ascertain accurate estimation of risk predictors and our model was confirmed in the validation cohort, but external validation is warranted by further studies. Secondly, presence of liver cirrhosis was not rigorously screened before study enrollment or during follow-up. It would be not feasible to screen all diabetic patients with liver ultrasound in outpatient settings. Since our HCC prediction model utilized commonly available parameters from routine practice of diabetes, our data may give clue to the selection of high risk diabetic patients for ultrasound screening for HCC. Thirdly, anti-HBc data was not available in most of our patients. Although HBsAg positive patients were excluded in our cohort, occult hepatitis B virus infection may have contributed to HCC development, considering high hepatitis B virus prevalence in Korea before introduction of national vaccination program. Finally, genetic risk markers were not studied in our analysis. Since PNPLA3 single nucleotide polymorphism is reported to promote HCC risk in NASH[47], incorporation of genetic data may improve performance of our model for HCC prediction.

In conclusion, old age, low serum TG levels and high serum GGT levels may predict risk of HCC in diabetic patients who do not have chronic viral hepatitis or alcoholic cirrhosis. Scoring system using these parameters may be useful in identifying subgroups of the diabetic population who are at an increased risk of HCC.

## Supporting Information

S1 Fig Correlation between serum triglyceride levels and fibrosis marker in derivation and validation cohorts. Pearson’s correlation analysis showed that negative correlation existed between serum triglyceride levels and FIB-4 scores in both derivation and validation cohorts.
(TIF)

S1 Table Characteristics of HCC detected during study period. (DOCX)

S2 Table DM-HCC risk score. (DOCX)
