# Supplementary figures and images for: Predictors of Increased Risk of Hepatocellular Carcinoma in Patients with Type 2 Diabetes
Source: PLoS One. 2016 Jun 30;11(6):e0158066. doi: 10.1371/journal.pone.0158066 (PMC4928920; doi:10.1371/journal.pone.0158066)

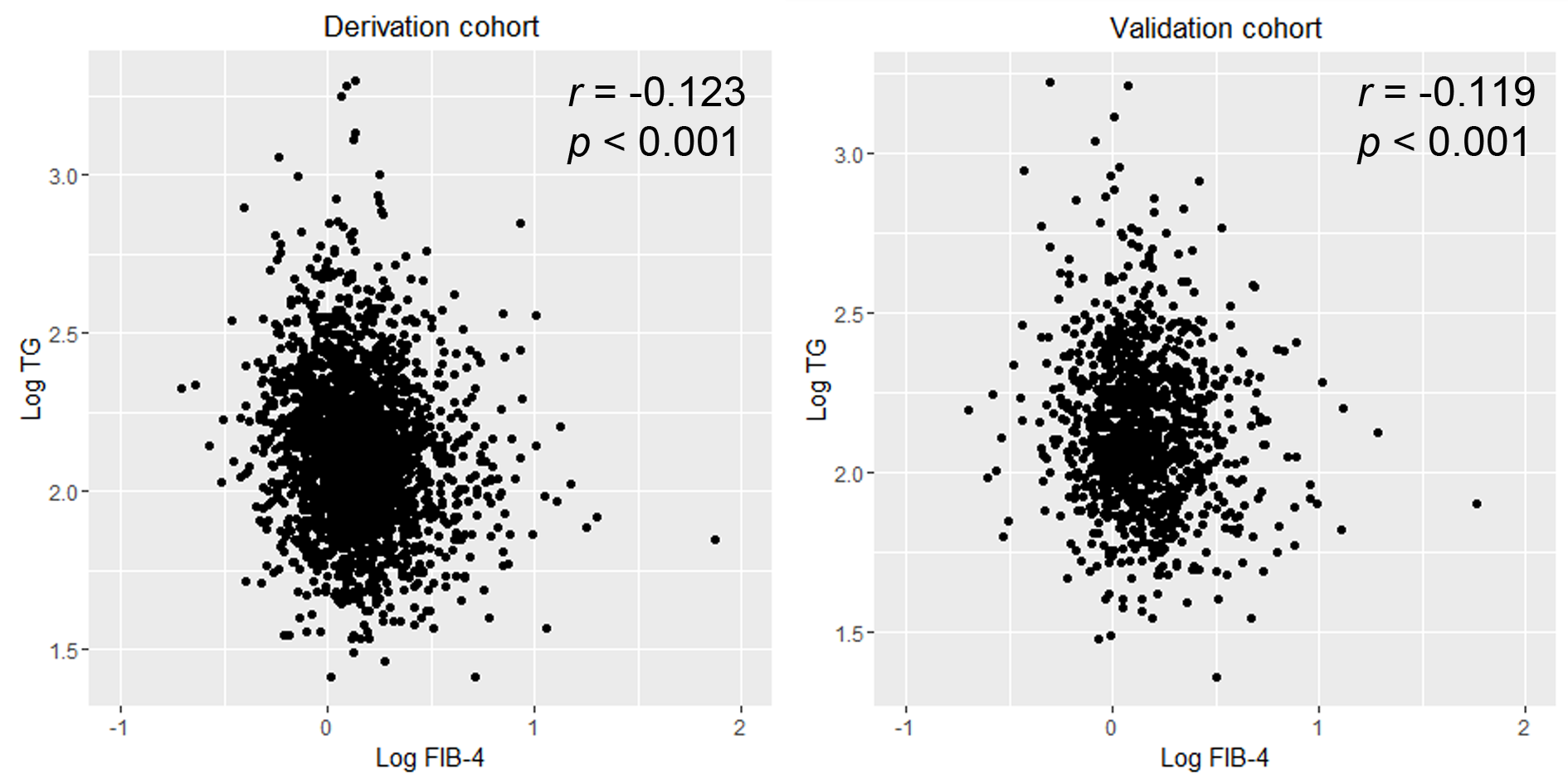

Supplement: S1 Fig — Pearson’s correlation analysis showed that negative correlation existed between serum triglyceride levels and FIB-4 scores in both derivation and validation cohorts. (TIF) [file pone.0158066.s001.tif]
