# Supplementary material for: Predictors of Increased Risk of Hepatocellular Carcinoma in Patients with Type 2 Diabetes
Source: PLoS One. 2016 Jun 30;11(6):e0158066. doi: 10.1371/journal.pone.0158066 (PMC4928920; doi:10.1371/journal.pone.0158066)
Supplement: S1 Table — (DOCX) [file pone.0158066.s002.docx]

**S1 Table.** Characteristics of HCC detected during study period

| Variables | HCC (n=36) |
| --- | --- |
| Age, years at diagnosis | 77.4 (59-95) |
| Male, no. (%) | 30 (83.3) |
| Max size of HCC, cm | 5.5 (1-20) |
| Number of HCC | 1.5 (1-3) |
| Portal vein invasion, no. (%) | 5 (13.9) |
| Distant metastasis, no. (%) | 3 (8.3) |
| Stage (I/II/IIIA/IIIB/IIIC/IVA/IVB), % | 38.9/5.6/13.9/11.1/5.6/16.7/8.3 |

Variables are expressed as median (range) or numbers (%).
