# Supplementary material for: Predictors of Increased Risk of Hepatocellular Carcinoma in Patients with Type 2 Diabetes
Source: PLoS One. 2016 Jun 30;11(6):e0158066. doi: 10.1371/journal.pone.0158066 (PMC4928920; doi:10.1371/journal.pone.0158066)
Supplement: S2 Table — (DOCX) [file pone.0158066.s003.docx]

**S2 Table.** DM-HCC risk score

| Parameter | Score |
| --- | --- |
| Age, years |  |
| $\mathbf{>}$65 | +11 |
| $\boldsymbol{\leq}$ 65 | 0 |
| GGT (IU/L) |  |
| $\mathbf{>}$80 | +16 |
| 41 – 80 | +8 |
| $\boldsymbol{\leq}$40 | 0 |
| TG (mg/dL) |  |
| $\mathbf{<}$ 150 | +6 |
| $\boldsymbol{\geq}$ 150 | 0 |
